# Global Warming and Dairy Cattle: How to Control and Reduce Methane Emission

**DOI:** 10.3390/ani12192687

**Published:** 2022-10-06

**Authors:** Dovilė Bačėninaitė, Karina Džermeikaitė, Ramūnas Antanaitis

**Affiliations:** Large Animal Clinic, Veterinary Academy, Lithuanian University of Health Sciences, LT-47181 Kaunas, Lithuania

**Keywords:** global warming, methane emission cattle, cattle, rumination, chewing activity, methane emission, feed additive, rumen microbiome

## Abstract

**Simple Summary:**

Concerns about greenhouse gas (GHG) emissions from livestock and dairy farms, as well as their connection to global warming and climate change, have grown among the general public worldwide in recent years. To evaluate these emissions, there is a need to use reliable methods. Enteric methane (CH_4_) and other greenhouse gas emissions from ruminants can be mitigated in numerous ways. The objectives of this review were to examine currently available knowledge about methane evaluation and mitigation strategies, and food supplements. We wanted to present a critical view and raise visions of what is known and unknown about GHG reduction and control.

**Abstract:**

Agriculture produces greenhouse gases. Methane is a result of manure degradation and microbial fermentation in the rumen. Reduced CH_4_ emissions will slow climate change and reduce greenhouse gas concentrations. This review compiled studies to evaluate the best ways to decrease methane emissions. Longer rumination times reduce methane emissions and milk methane. Other studies have not found this. Increasing propionate and reducing acetate and butyrate in the rumen can reduce hydrogen equivalents that would otherwise be transferred to methanogenesis. Diet can reduce methane emissions. Grain lowers rumen pH, increases propionate production, and decreases CH_4_ yield. Methane generation per unit of energy-corrected milk yield reduces with a higher-energy diet. Bioactive bromoform discovered in the red seaweed *Asparagopsis taxiformis* reduces livestock intestinal methane output by inhibiting its production. Essential oils, tannins, saponins, and flavonoids are anti-methanogenic. While it is true that plant extracts can assist in reducing methane emissions, it is crucial to remember to source and produce plants in a sustainable manner. Minimal lipid supplementation can reduce methane output by 20%, increasing energy density and animal productivity. Selecting low- CH_4_ cows may lower GHG emissions. These findings can lead to additional research to completely understand the impacts of methanogenesis suppression on rumen fermentation and post-absorptive metabolism, which could improve animal productivity and efficiency.

## 1. Introduction

Carbon dioxide (CO_2_) and methane are the two most important greenhouse gases, and since 1950, their concentrations in the atmosphere have increased from 350 to 410 ppm (a rise of 28%) and from 1100 to 1875 ppb (a rise of 70%), respectively [1]. About 24% of worldwide methane emissions and a much greater fraction of anthropogenic methane emissions are related to the production of fossil fuels (coal, oil, and natural gas) [2]. Human activities, including growing rice, keeping ruminant animals, using landfills and compost, treating wastewater anaerobically, producing natural gas, and mining coal, account for more than 60% of all CH_4_ emissions. Wetlands and oceans account for the remaining 40% of methane emission [3]. After livestock, rice cultivation is the largest source of methane. Flooded-field-grown rice emits twice as much greenhouse gas than wheat [4]. Concerns have been raised in the realm of agricultural production regarding the effects that an increase in rice production may have on the surrounding environment, particularly regarding the emissions of greenhouse gases.Rice paddies are responsible for a significant amount of greenhouse gas emissions, specifically approximately 30 percent of all methane (CH_4_) and 11–25 percent of all nitrous oxide (N_2_O) emissions [5]. Rice paddies are thought to be one of the largest human-made sources of carbon monoxide in the air, with an estimated 11% of all human-made CH_4_ emissions coming from them [6]. Linquist et al., in their study, found out that in terms of area, the global warming potential (GWP) of CH_4_ and N_2_O emissions from the rice paddies was much greater than that of wheat or maize [7].

The agricultural sector is rapidly participating in greenhouse gas emissions. Globally in recent years, there has been a rising public concern about farm animals, dairy farms’ greenhouse gas emissions, and their impact on global warming and climate change [8]. Research has found that increased CH_4_ emissions can be substantially attributed to animal farming [3]. Manure decomposition and microbial fermentation in the rumen produce methane, wherein the animal expels from the rumen via eructation [9,10].

**Figure 1:**
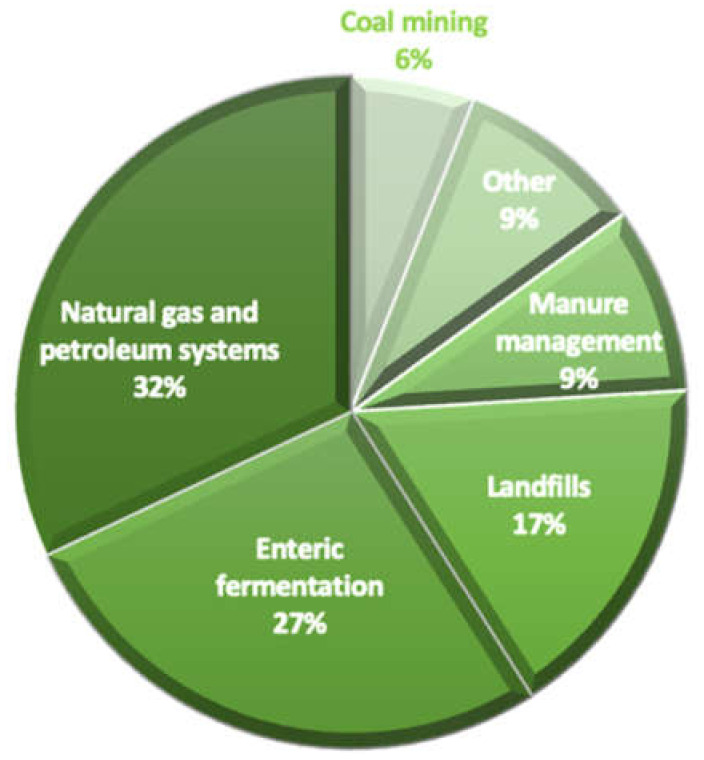
Source of US CH_4_ emissions in 2020.

In terms of CO_2_ equivalents, enteric fermentation and manure management emissions account for approximately 41% of agriculture’s overall GHG emissions [11]. Emissions of greenhouse gases from milk production account for over 70% of all GHG emissions before the farm gate, with enteric CH_4_ accounting for 35–55% of all farm emissions [12]. According to the United States Environmental Protection agency, enteric fermentation expels about 27% of all US CH_4_ emissions [13] (Figure 1). The investigation of nutritional and management strategies to minimize methane emissions is essential for long-term milk production [14,15,16]. Dairy cow milk output has increased dramatically in recent years due to improved selection, feeding, and herd management approaches [14]. Herbivores use their gut microbiota to convert fibrous feed resources into high-quality proteins (meat, milk) for human consumption [17].

A lot of study is ongoing to figure out how to reduce ruminant enteric methane emissions. There is no doubt that feeding contributes to methane release in dairy cattle, as it is produced during the digestion of high-fiber diets [18]. Some mitigating strategies lower pasture digestion or feed consumption, which can affect feed conversion ratio and methane emissions per kilogram of product [10]. A range of dietary management measures has been explored in order to lower enteric methane generation. Dropping diet forage to concentrate ratios, incorporating rumen modifications and methane antagonists like bromoform or other phytocompounds in the diet, or increasing dietary oil content are all nutritional alternatives for methane mitigation [10,19,20]. A high-fiber diet can promote acetate production. The synthesis of acetate and butyrate is followed by the release of metabolic hydrogen, which has a deleterious impact on microbial development and on feed digestibility while accumulating in rumen fluid [10,21]. Some food additives can be effective in the laboratory but not in reality [22]. The use of naringin and chitosan positively affected fermentation patterns, increasing propionic acid while reducing acetate and methane production by 12% and 31%, respectively. Still, for the in vivo trial where chitosan and naringin were administered either separately or in a combination given directly into the rumen, both additives did not show a positive effect on rumen fermentation or enteric methane production [22]. Other authors have studied seaweed‘s impact on methane emissions. Kinley et al. investigated *Asparagopsis taxiformis*. The study showed in vitro that 20 g/kg of fodder with the mentioned algae almost completely abolished CH_4_ generation while having no detrimental impact on forage digestibility [23]. Using oil as a feed supplement also can give great expectations. Lipids can suppress methanogenesis by substituting rumen fermentable organic matter in the diet and by biohydrogenating unsaturated fatty acids, reducing the number of ruminal methanogens and protozoa [24]. To meet future global demands, the livestock industry must investigate natural feed additives that improve nutrient utilization efficiency, provide antibiotic alternatives, and reduce ruminant methane emissions.

To evaluate methane emissions, there is a need to use reliable methods. Garnsworthy et al. compared various different methane measurement methods [10]. In the research, methods like respiration chambers, the SF6 tracer technique, milking or feeding breath sampling, the GreenFeed^®^ (GF) system (C-lock Inc., Rapid City, SD, USA), and the laser methane detector were compared. The study’s purpose was to evaluate and compare the suitability of various technologies for measuring methane on the herd or individual animal level [10]. When individual cows on commercial farms can be reliably measured directly for enteric CH_4_, it allows for more focused emission mitigation. It also provides the potential for farm-level benchmarking and the selection of cows with low enteric CH_4_ production. The use of mobile gas analyzers to detect CH_4_ emissions from large numbers of animals across populations is of great interest [9,10].

To slow climate change and lower greenhouse gas concentrations in the atmosphere, CH_4_ emissions must be reduced. There is a need to perform more studies to find the most effective food supplement or its composition and contribute to reducing methane emissions without compromising animal health and production. This demands the use of low-cost and portable technologies for estimating CH_4_ emissions on a wide scale while combining it with trustworthy forage [25,26].

The review examines currently available knowledge, its gaps, and the prospects for enteric CH_4_ mitigation in the future. We needed to give a critical view of what is known and what is unknown to raise visions, goals, and challenges for future scientists, governments, manufacturers, and the livestock industry.

## 2. Role of Dairy Cattle in Global Warming

The atmosphere contains natural greenhouse gases such as carbon dioxide, methane, water vapor, and nitrous oxide (N_2_O), as well as synthetic greenhouse gases such as hydrofluorocarbons (HFCs), perfluorocarbons (PFCs), chlorofluorocarbons (CFCs), and sulfur hexafluoride (SF_6_) [13]. Agricultural systems are a substantial source of GHG emissions into the atmosphere, accounting for around 30% of total anthropogenic emissions, including indirect emissions through land-cover change, as CO_2_, CH_4_, and nitrous oxide are the three principal greenhouse gases released by animal production [27]. Animal husbandry is a substantial source of GHGs, accounting for 14.5 percent of world emissions, which is roughly the same as the transportation industry [28]. Ruminant livestock is expected to emit between 80 and 95 million tonnes of CH_4_ per year globally [29,30,31]. CH_4_ generation also represents a loss of energy availability to the host ruminant animal, often accounting for between 2% and 12% of total energy availability [25]. Cattle and sheep production systems contribute the most to GHG emissions in agriculture, accounting for up to 18% of total global GHG emissions, mostly in the form of enteric methane [32]. Enteric CH_4_ emissions from ruminant production are the most common source of greenhouse gases, accounting for 46 percent for dairy and 55 percent for small ruminant productions of total CO_2_e emissions [33]. Cattle are commonly mentioned among food-producing animals due to their significant contribution to the sector’s GHG emissions, particularly methane [34]. The enteric fermentation process provides more than 90% of CH_4_ emissions from livestock and 40% of agriculture GHG emissions [35]. According to the Intergovernmental Panel on Climate Change and Food and Agriculture Organization of the United Nations—a fully developed cow can emit up to 500 liters of methane each day, which accounts for approximately 3.7 percent of all greenhouse gas emissions [36]. Almost all the methane is formed in rumen while using protective mechanisms and released by burping. The rumen is a complex system comprised of elements like protozoa, bacteria, archaea, viruses, fungi, and bacteriophages, all of which contribute to the harvesting of food energy and subsequent provision of nutrients to the host. CH_4_ is produced as a by-product of this fermentative process when hydrogen is liberated and used by methanogens to form CH_4_ [37,38,39,40]. Rumen *Archaea* are microorganisms that produce methane and water by combining metabolic hydrogen and carbon dioxide. *Archae* also has a role in saving rumen from excess hydrogen by producing methane [10]. The number of fiber fractions digested in the rumen is proportional to the rumen metabolism product amount. The more fiber content an animal digests, the more methane will be produced because of the acetate and hydrogen amounts in the rumen [10]. That shows that the rumen environment can influence methanogen production [15,41].

## 3. Measurement and Estimation of CH_4_

As indicated by frequent reviews, a wide spectrum of technologies (Table 1) is being developed and used to quantify methane emissions by individual dairy cattle under varied environmental conditions [10,25,42]. All approaches have various application scopes, benefits, and drawbacks, and none are excellent [9]. Due to the availability of portable gas analysis equipment and the discovery that frequent methane emission measurement during robotic milking has a high correlation with respiration chamber measurements of total methane production from the same cow, a sniffer or breath sampling to measure enteric methane emissions from individual cows has shown great prospects [10,43,44]. Potential causes of error, such as the cow’s head position and the number of measurements collected, must be considered [45]. There also is a need to have sensors such as a proximity sensor to identify the location of the cow’s head while making gas spot samples [45]. Different techniques analyze separate aspects of methane release. Oral, nasal, and anal emissions can only be measured in a respiration chamber. In other approaches, anus emissions are not considered, and only methane emitted in breath is measured. It is necessary to take breath measurements since 99 percent of methane is expelled through the mouth and nose, whereas just 1 percent is emitted through the anus [10,46]. Portable and noninvasive methods that do not disrupt the cow’s daily routine or environment are particularly interesting [9,25].

### 3.1. Methane Prediction Models

Aside from studies on methane emission prevention, significant research emphasis has shifted to the creation of prediction models of methane emissions from livestock, as global warming reduces agricultural production. An accurate estimate of enteric methane generation from ruminants can help to balance increased animal production with the environmental consequences [61]. Methane emissions can be quantified as units of methane per animal per day, dry matter intake, or metabolic body weight (MBW) per day. There are many models from various studies. Storlien et al. and Niu et al. created a database for the basic models that show us CH_4_ production, DMI, and contents of EE or FAs and NDF in diets for dairy cows and roughage composition [62,63]. A shortened version of the authors’ database is presented in Table 2. Moreover, there are some prediction models based on g CH_4_/per animal/d (Animal-based models), g CH_4_/kg DMI (DMI-based models) (Table 1), and g CH_4_/kg metabolic bodyweight/d (MBW-based models) []. Prediction models for methane generation may be divided into two types: statistical models and dynamic models. Dynamic models include extensive digestive and rumen fermentation mechanisms to simulate and forecast methane generation. Ideally, these models can imitate system dynamics at lower levels of aggregation and can forecast a wider range of eventualities. Because of the multiple inputs and computing needs, dynamic models are difficult to apply to realistic predictions [61,63,64].

**Figure 2:**
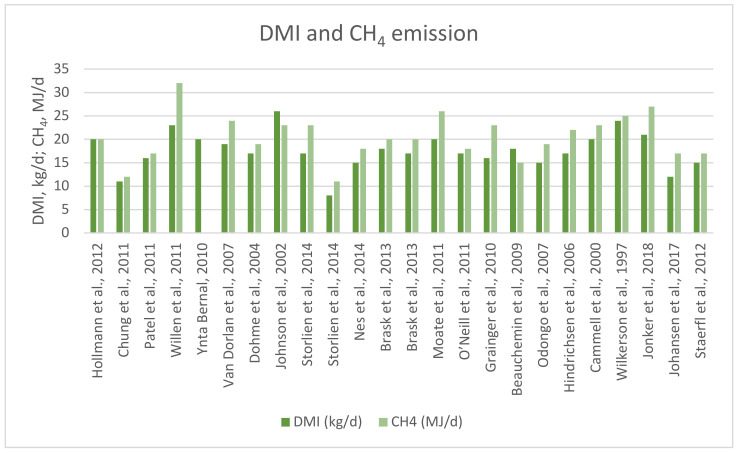
CH_4_ emission MJ/d compared with DMI [62,63].

DMI and CH_4_ production had a substantial positive connection, suggesting that as a dairy cow eats more feed, more CH_4_ is generated due to increased substrate availability for microbial fermentation [63] (Figure 2). The findings of the Niu et al. study analyzed the influence of explanatory factors on the variability of CH_4_ production among areas. When all other variables were held constant, the slopes of DMI to CH4 production varied from 13.0 to 15.3 g of CH_4_/kg of DMI for the EU cows. The equivalent values for US cows were lower, ranging from 11.3 to 12.3 g of CH_4_/kg of DMI [63]. Another study found a 2.1% decrease in Ym per kg DMI rise from dairy cows [65].

**Table 2:** Methane prediction models database.

Lactation Stage	Roughage	Concentrate	DMI (kg/d)	CH_4_ Collection Technique	CH_4_ (MJ/d)	References
L	Corn silage	Ground corn	20	Room tracer approach	20 (14–26)	[66]
NL	Grass hay or barley silage	Barley grain	11	Sulfur hexafluoride tracer gas technique	12 (11–17)	[67]
L	Grass silage	Oats, barley, peas and rapeseed cake	16	Sulfur hexafluoride tracer gas technique	17 (16–18)	[68]
L	Grass silage	Barley, wheat and maize	23	Sulfur hexafluoride tracer gas technique	32 (28–36)	[69]
L	Grass silage	Barley, wheat and oats	20	Sulfur hexafluoride tracer gas technique	26 (24–28)	[70]
L	Ryegrass, white and red clover	Pelleted barley	19	Chamber	24 (23–26)	[71]
L	Grass and maize silage	Barley	17	Chamber	19 (17–21)	[72]
L	Alfalfa hay and alfalfa silage	Barley, corn and peas	26	Room tracer approach	23 (22–25)	[73]
L	Grass silage	Barley	17	Sulfur hexafluoride tracer gas technique	23 (20–29)	[62]
NL	Grass silage	Wheat starch (non-NDF concentrate)	8	Sulfur hexafluoride tracer gas technique	11 (10–12)	[62]
L	Grass silage	Wheat starch (non-NDF concentrate)	15	Sulfur hexafluoride tracer gas technique	18 (17–19)	[74]
L	Grass and corn silage	Rapeseed meal, rapeseed cake, cracked rapeseed and rapeseed oil	18	Sulfur hexafluoride tracer gas technique	20 (17–23)	[75]
L	Grass silage and maize silage	Rapeseed meal, whole crushed rapeseed	17	Sulfur hexafluoride tracer gas technique	20 (18–22)	[76]
L	Alfalfa hay and ryegrass silage	Cracked wheat grain	20	Chamber	26 (25–28)	[77]
L	Corn and grass silage	Soybean meal and rolled barley	17	Sulfur hexafluoride tracer gas technique	18 (14–22)	[78]
L	Corn silage and alfalfa haylage	Cracked wheat grain	16	Sulfur hexafluoride tracer gas technique	23 (21–25)	[79]
L	Barley silage	Steam rolled barley and pelleted supplement	18	Chamber	15 (13–16)	[30]
L	Haylage, corn silage and high	Corn gluten and soybean meal	15	Head hood	19 (15–23)	[80]
L	Hay, grass and corn silage	Barley and wheat bran	17	Chamber	22 (18–24)	[81]
L	Corn and grass silage	Rapeseed meal, sunflower meal, ground wheat and maize gluten feed	20	Chamber	23 (22–23)	[82]
L	Alfalfa silage High moisture corn and	High moisture corn and dry corn	24	Chamber	25 (24–26)	[83]
L	Ryegrass, white clover, or mature, diverse pasture	0	21	Greenfeed system	27 (26–28)	[84]
L	Grass clover silage	0	12	Chamber	17	[85]
L	Ryegrass	0	15	Chamber	17 (16-19)	[86]

### 3.2. Biomarkers for Controlling of GHG

#### 3.2.1. Rumination Time

Rumination affects the entire digestion process, including feed passage rate, free feed consumption in dairy cows, and the cow’s milk performance [15,87,88]. Watt et al. demonstrated that increasing rumination time enhances feed intake and milk output [89]. Longer ruminating times are linked to decreased methane emission, and lower methane release per milk unit in high-yielding dairy cows fed a maize silage-based partial mixed feed without access to pasture [15]. Mikula et al. show that low rumination cows generated 1.8 percent more CH_4_ than medium rumination cows and 4.2 percent more than high rumination cows, resulting in the highest daily CH_4_ output. Cows in the high rumination group produced 2.9 percent less CH_4_ per milk unit than cows in the medium rumination group and 4.6 percent less CH_4_ than cows in the low rumination group [15]. In addition to aiding in the digestion of feed particles, rumination also helps to enhance saliva production [15,89,90]. Cow health and methane emission are connected through the rumen fluid pH and saliva production during the rumination period [15]. However, some studies show no relation between methane and rumination. Zetouni et al. identified no link between ruminating time and methane emission in high-yielding dairy cows [91].

#### 3.2.2. Rumen Microbiome

Methanogens are found in various sites inside the rumen, including the epithelium, biofilms, protozoa, and fungus [92]. Saliva lysozyme plays a crucial role in the rumen microbiota by limiting the proliferation of Gram-positive bacteria. This can also affect the selection of methanogenic microbes, change the rumen environment, and modify methane emissions [15]. The concentration of dissolved hydrogen used in methane production can be affected by an increase in the acetate and butyrate content in rumen fluid [15,19]. In the rumen microbial ecology, ciliate protozoa are major H2 producers that play an important role in interspecies H_2_ transfer and CH_4_ emissions. Protozoa have been shown to have a strong correlation with CH_4_ emissions, which suggests that protozoa could be a potential target for CH_4_ mitigation [92,93,94]. Reduced neutral detergent fiber from forage (NDF) and increased concentrate intake may be associated with a lower rumen pH. An increase in propionate and a decrease in acetate and butyrate levels can decrease hydrogen equivalents that would be turned to methane and are antagonists of methanogenesis [15,39].

### 3.3. Methane Emissions and Animal Performance in Dairy

An analysis of lactating of Holstein–Friesian, Jersey, and cannulated dairy cows fed a high-quality dairy feed supplemented with silage or high-quality fodder performed by Min et al. showed that milk production has a significant relationship with methane production [92]. The relationship between milk production and methane production in a grain-based diet was not meaningful, but a significant difference was found when comparing CH_4_ emissions per kg in cattle given grain- and forage-based diets (R2 = 0.38 − 0.40). By adding grain to the feed diet, the starch concentration is increased. It decreases crude fiber content, lowers rumen pH, and stimulates propionate formation in the rumen while decreasing CH_4_ yield [92,95]. Thus, food quality and components significantly affect CH_4_ production: poor feed quality results in increased CH_4_ production. This is the largest source of cow energy loss, and avoiding it is crucial for increasing average daily gain (ADG) or milk output. However, increasing productivity through the use of high-grain diets must be weighed against the expense of feed production, fertilizer use, and machinery use, all of which increase fossil fuel consumption and N_2_O emissions [92]. High-quality grain-based diets provide more energy for animal production as a percentage of the GEI or DMI (kg/d) and dilute maintenance costs more than low-quality forage-based diets or grazing, resulting in a lower CH_4_ g/kg ECM. Min et al. and Knapp et al. also discovered that CH_4_ g/d decreased (*p* 0.001; R2 = 0.46) as ECM, g/kg, increased in dairy cattle. As a result, enteric CH_4_ emissions per unit of energy-corrected milk (ECM) (CH_4_/ECM) are important indicators of biological, nutritional, and environmental quality, as well as economic activity [19,92].

## 4. Methods to Reduce GHG

### 4.1. Feed Supplements

Several diets of various compositions to help reduce methane emissions have been investigated over many studies in the past few years. Some studies analyze grass silage-based feeding starting with an earlier harvest of grass and ending with various supplements [30]. Some authors suggest that replacing grass silage (GS) with maize silage (MS) promotes greater propionate rather than acetate fermentation in the rumen, lowering CH_4_ production in dairy cows [30]. When maize silage totally replaced grass silage in the diet of dairy cows, a reduction was noticed in CH_4_ emissions of between 8% and 11% [96]. Aguerre et al. discovered a reduction in methane per energy-corrected milk production when non-fiber carbs were increased in the diet by increasing concentrate intake from 32% to 53% [97]. Knapp et al. also found that diets with higher energy or greater digestibility can reduce methane output per energy-corrected milk yield [19]. Several innovative treatments, such as dietary supplementation with algae, phytocompounds like saponins and tannins, and essential oils, may help to reduce CH_4_, although further research is needed. Methane-reducing supplements and their impact on animal health and sustainability are discussed in Table 3.

#### 4.1.1. Algae, Bromoform

Seaweeds are among the world’s fastest-growing photosynthetic organisms and absorb considerable amounts of carbon dioxide and nutrients such as nitrogen, phosphorus, and heavy metals from the water in which they are grown [120,121]. Globally, it is believed that seaweeds absorb roughly 200 million tonnes of CO_2_ annually. As algae die, a significant portion of the carbon stored in their tissues is carried to the deep waters. However, these natural carbon sinks are also threatened by global warming. [122].

Algae are rich in nutrients and bioactives, such as proteins, carbohydrates, and, to a lesser extent, lipids, and are full of peptides, saponins, alkaloids, and pigments. Some algae include phlorotannin and bromoforms, which are halogenated chemicals that block the cobamide-dependent coenzyme M during methanogenesis [123]. In vitro screening of 20 tropical marine macroalgae species by Machado et al. revealed that the genus *Dictyota* (brown algae) and genus *Asparagopsis* (red algae) have the greatest potential for methane emitting [124]. The bioactive bromoform present in the red seaweed species *Asparagopsis taxiformis* has been identified as an agent capable of considerably reducing enteric methane generation in livestock because of bromoform’s ability to impede its biosynthesis of it [37,124]. A 67% reduction in methane was found in one study when *Asparagopsis armata* was provided a 1% inclusion in a feed for lactating dairy cows, with no residues found in milk. Another trial with confined cattle revealed that adding 0.2% of organic feed matter in the form of dried *Asparagopsis* reduced methane emissions up to 98% and enhanced weight gain by 42% with no detrimental effects on feed intake or rumen function [23,100]. Similar reductions in methane production were observed in dairy cows that were fed 0.5 percent dry matter of *Asparagopsis taxiformis*, ranging from 55% to 80% [125]. Another study’s in vivo results showed that cows’ methane production dropped significantly by 26.4% at a low (0.5%) and 67.2% at a high (1%) level of *Asparagopsis armata* inclusion and bromoform concentration in milk, which was not significantly different between treatments [126]. A sheep study revealed that feeding up to 3% *A. taxiformis* to sheep reduced methane production in a dose-dependent way over a 72-day period, with an 80% reduction at the highest dose and no changes in body mass increase [127]. Moreover, sheep that were fed *Asparagopsis* showed much lower levels of total volatile fatty acids and acetate but greater levels of propionate. There were no differences in live weight gain. The fact that methane emissions did not rise over time suggests that the rumen methanogen population did not adapt to the algae [127]. Additionally, a diet with *Asparagopsis* supplementation can cause ruminal mucosa changes [127]. This leads to the conclusion that further studies on algae are required. Bromoform is damaging to the environment and can harm human health. Furthermore, a life cycle evaluation will need to look at the CO_2_ emissions from growing, harvesting, drying, and shipping algae, which could outweigh any reductions in CH_4_ emissions from ruminants [127].

##### Algae Cultivating and Sustainability

Over the past seventy years, seaweed farming technology has advanced dramatically in Asia and, more recently, in the Americas and Europe [128]. Most cultured seaweeds are presently utilized for human consumption, either directly or as additives (hydrocolloids like agar, alginates, and carrageenan’s) (90% of production) [102]. The annual global production of seaweeds continued to increase in 2016, reaching 31.2 million tonnes in fresh weight. Only 3.5% of this was gathered from native populations, while 96.5% was produced in aquaculture, accounting for 27% of the world’s total aquaculture production [128]. According to FAO, algae production comprised 35,1 million tonnes in 2020 [129]. Unfortunately, the life cycle of *A. taxiformis* could not be closed, but useful procedures for the collection of wild species and better methods for the germination of carpospores must be developed. The primary objective of seaweed aquaculture is to balance the positive and negative components of the growing system to ensure that the environment is not severely impacted and the ecological system’s status quo is not drastically disrupted [128]. According to Nilsson et al., under the default scenario, the GHG emissions from seaweed agriculture were 9.2 kg 5CO_2_e kg1 seaweed. The addition of salt (NaCl) to the inoculum tank’s water to raise the salinity and improve the seaweed’s growth was responsible for 48% of the stage’s total GHG emissions. Several other types of impacts, such as the depletion of resources, marine eutrophication, and water usage, can be traced back to the salt influx as well. The scenario study revealed that switching from rock salt (used in the baseline scenario) to sea salt greatly reduced greenhouse gas emissions [130]. CO_2_ mitigation can benefit from the harvesting of algae for use in biofuels and other sectors (food, feed, medicines, and fertilizers) [128].

Seaweed farming is possible offshore, onshore, and even in integrated aquaculture systems. The cultivation of seaweed is determined by the species, farm location, and cultivation infrastructure. Due to the impact of abiotic and biotic factors, the current onshore and offshore farming techniques are not yet environmentally sustainable and are economically unstable, as production changes quite rapidly [102,128]. Most seaweeds are grown near the water’s surface in order to get enough sunlight for photosynthesis; thus, they are typically grown in nearshore regions for operational and logistical reasons. Nearshore activities are typically less costly in terms of investment and operating costs. However, a number of factors, including competition for nearshore areas from urban development, recreation, fishing, fish farming, and/or other activities, pollution in nearshore waters, and rising seawater temperatures, pose constraints or challenges to seaweed cultivation in the nearshore areas. Cultivating seaweeds further offshore can assist in overcoming nearshore limits, and seaweed agriculture might be linked with other offshore operations such as wind energy generation. However, seaweed cultivation in the open ocean faces technical feasibility challenges (waves, deep-water farm sites), economic viability, and sustainability [131]. Offshore, onshore cultivation methods are less costly and labor-intensive for the maintenance of seaweeds than land-based ones. Due to the minimal installation and maintenance cost, connecting seaweeds to ropes, lines, or nets is a common cultivation method. In these farming systems, the susceptibility of the structures and seaweeds to the harshest ocean and environmental conditions is a serious concern. To limit environmental risk to the crop and ensure economic viability, farms must be expansive and located in a variety of locations. There is a need for a multi- and inter-disciplinary team to optimize aquaculture to mitigate the risks associated with seaweed farming and promote the development of new and improved aquaculture systems and seaweed quality [128]. Beyond the realm of traditional aquaculture, the cultivation of seaweed could serve as a general tool for circular resource management, the treatment of wastewater produced by land-based farming and municipal treatment plants, the biosorption of heavy metals, and the recolonization of artificial reefs [132]. Despite this predicted increase, the farming system optimization that ensures a steady supply of seaweed and all its constituents is still in its infancy [128]. Biosecurity threats from exotic species, consumer risks from heavy metals and pollutants, diseases, and potential ecosystem impacts such as the shading of seagrass beds below poorly located farms, and co-opting of nutrients necessary for the normal function of neighboring ecosystems should all be taken into account when developing sustainability standards [102,133].

#### 4.1.2. Phytocompounds: Polyphenolic Substances (Tannins, Saponins), Essential Oils, Flavonoids

Plant components such as essential oils, tannins, saponins, and flavonoids have been studied for their anti-methanogenic activities. Cobelis et al. reviewed that essential oils extracted from thyme, garlic, eucalyptus, oregano, or cinnamon showed methanogenesis-reducing properties in vitro. Still, just a few have been found to have long-term anti-methanogenic effects in vivo [134]. Manh et al. show that eucalyptus leaf meal addition at 100 g/d for grazing animals could be an alternative feed booster: it reduces the development of rumen methane gas in cattle while preserving mineral digestion [117]. Oregano and white thyme essential oils can modulate ruminal fermentation and decrease rumen methanogenesis without affecting feed digestibility, showing promise as alternatives to ionophores for methane reduction in beef cattle [119,135]. Moreover, it was studied that cinnamon and cloves have a phenolic monoterpene that demonstrated antimicrobial activity against both Gram-positive and Gram-negative [136]. Coriander oil can regulate in vitro digestibility and CH_4_ generation [137]. Jayanegara et al. also reviewed that, according to many studies, condensed and hydrolyzable tannins also show promise for mitigating CH_4_ emissions [113]. Tannins can decrease methane synthesis in the rumen either directly or indirectly by inhibiting methanogens or protozoa [104]. Methane reaction to tannin feeding varies greatly depending on the tannin source, kind, and molecular weight, as well as the methanogenic community present in the animal. A 30 in vitro and in vivo meta-analyses revealed that increasing tannin levels reduced CH_4_ generation expressed relative to digestible organic matter [113]. Another tannin study showed that cows produced less methane as the amount of *Leucaena* consumed climbed from 0 to 36% of diet DM when fed a low-quality tropical grass (*Megathhyrsus maximus*) and increasing amounts of chopped legume leaf from *Leucaena leucocephala*. Methane emissions were estimated by open-circuit chambers [138]. Additionally, other tropical legumes, such as *Desmanthus spp.*, have also been shown to reduce intestinal CH_4_ emissions in cattle grazing in tropical grasslands [139]. Compounds like saponins are naturally occurring detergents found in numerous plants. The use of saponin-containing plants as a potential method for reducing or eradicating protozoa in the rumen has gained popularity. Reduced populations of ruminal ciliate protozoa may accelerate microbial protein flow from the rumen, increasing feed utilization efficiency and decreasing methanogenesis [112]. Many saponin sources were investigated like Quillaja (*Quillaja saponaria*), *Gypsophilla paniculata*, *Tribulus terrestris*, Tea (*Camellia sineis*), Yucca (*Yucca schidigera*) [140,141,142]. Jayanegara et al., in their review, calculated that increases in the concentration of a saponin-rich source resulted in a decrease in the amount of CH_4_ emitted per unit of substrate incubated with a curvilinear pattern (*p* < 0.05). The study showed that when administered at a concentration of around 500 mg/g DM, saponin-rich sources had no effect on lowering the relevant CH_4_ parameter. When expressed in milliliters per 100 milliliters of total gas generated, increasing the concentration of the saponin-rich source lowered the CH_4_ linearly (*p* < 0.001). The protozoal count fell significantly (*p* < 0.05) at higher saponin levels. When several saponin-rich sources were compared, all saponin-rich sources, namely quillaja, tea, and yucca saponins, produced less methane per unit of total gas than the control (*p* < 0.05) [113]. Ku-Vera et al., in their review article, analyzed studies that found that a commercial citrus extract containing flavonoids (Bioflavex^®^) decreased methane production and the population of hydrogenotrophic methanogenic archaea while increasing propionate concentrations and the population of *Megasphaera elsdenii* [104,139,143]. Stoldt et al. discovered that glucrohamnoside of quercetin had no influence on the production of methane or the energy metabolism of Holstein cows [144]. Cui et al. showed that supplementing multiparous Chinese Holstein cows with rutin 3.0 mg/kg enhanced milk output (10.06 percent) over time and improved the dairy cow’s metabolism and digestibility [145].

##### Phytocompounds. Sustainability

Different plants need to be obtained in different ways. Although cinnamon can be used to reduce methanogenesis, it is considered moderately sustainable [146]. Cinnamon bark can be harvested at approximately the fifth year of the tree’s life. To obtain the cinnamon bark, harvesters cut down the whole cinnamon tree and peel away the outer bark to reach the inner bark [147]. When intercropped with other trees, cinnamon forests grow organically without the use of agricultural pesticides. Typically, cinnamon begins to regrow nearly immediately after being clipped [146]. Regarding water and carbon footprint, it takes 15,526 L of water to produce 1 kg of cinnamon and 1.6 kg CO_2_e to produce 1 kg of spices, equivalent to a car driving the equivalent of 6 km [146]. Garlic and oregano also can be seen as sustainable sources for reducing GHG. It takes 589 L of water to produce 1 kg of garlic and takes 7048 L of water to produce 1 kg of dried oregano [148]. Some studies show the sustainability of garlic cultivation. The result of the research showed that garlic cultivation has a status as sustainable, with a sustainability index value of 66.44 [149]. Another additive—Eucalyptus—is particularly characterized by sustainability. In some places, Eucalyptus is only allowed to grow to waist height before being harvested. The harvesting or distillation of Eucalyptus does not produce significant waste. The discarded leaves are returned to the furnaces as fuel or changed into garden mulch before being returned to the ground after oil extraction [150]. Eucalyptus trees may be harvested in as little as three to five years, making them a quickly renewable resource. Some kinds of eucalyptus can grow 4 m each year. In addition to their rapid growth, rapidly renewable plants also benefit the environment in other ways. They place far less strain on the ecology because they require less water and fertilizer than other plants [151].

#### 4.1.3. Oils: Rapeseed Oil

Few studies have demonstrated that low levels of lipid supplementation (4% of dietary dry matter intake) can reduce methane generation (up to about 20%) while enhancing the energy density of diets and benefiting animal productivity in some situations [24,79]. Including rapeseed oil (RSO) in the diet is another dietary option for efficiently decreasing enteric CH_4_ emissions in dairy cows, as Bayat et al. and Villar et al. [152,153]. When ruminal CH_4_ emissions and milk saturated fats are increased with plant oils in grass silage, the proportion of unsaturated fats and conjugated linoleic acid increases without altering digestibility, rumen fermentation, the number of rumen microbials or milk production, according to Bayat and colleagues [153]. Scientists found that supplementing nursing dairy cow diets with 5% RSO reduced CH_4_ emissions by up to 23%. In addition, Ramin et al. discovered that total methane emission (from breath and feces) was dramatically reduced when meals containing rapeseed oil were used. Total dry matter and nutrient consumption were reduced due to the mentioned oil supplementation [154]. Another study found that rapeseed oil supplementation reduced dry matter and nutrient intake, energy-corrected milk yield, milk fat and protein composition and yield, and general nutrient digestibility, except for crude protein. Oil in the diet reduced daily methane emission and intensity and enhanced the relative number of *Methanosphaera* and *Succinivibrionaceae* in the rumen while decreasing the abundance of *Bifidobacteriaceae*. In this investigation, dietary supplementation with 41 g rapeseed oil/kg in dry matter reduced daily CH_4_ emissions from lactating dairy cows by 22.5% [155]. Poulsen et al., in their in vitro study, added rapeseed oil to silage and observed a reduction in methane production related to a decrease of *Thermoplasmata (Methanomassiliicoccaceae)* and an increase in the relative abundance of both *Methanosphaera* and *Methanobrevibacter* [156]. Studies with lipid insertion to forage also showed that methane-producing *Methanosphaera* and *Methanobrevibacter* increased [157,158,159].

### 4.2. Reducing Greenhouse Gas Emissions through Genetic Selection

Genetically selecting low-methane (CH_4_) emitting cows can be an efficient and sustainable strategy for reducing GHG emissions from dairy cattle [160,161]. If we want to integrate CH_4_ into our breeding objectives, it is critical to understand the genetic relationships between CH_4_ traits and other economically significant traits. Several investigations conducted over the last decade have demonstrated that CH_4_ characteristics in dairy cattle have a low to moderate heritability, ranging from 0.11 to 0.33 [43,162]. Some studies were conducted using a multicountry database to estimate genetic parameters for methane features (Methane production MeP, Methane intensity MeI, etc.), as well as genetic correlations between methane traits and production, maintenance, and efficiency traits. The study showed that residual CH_4_ corrected for metabolic body weight (MBW) and energy-corrected milk (ECM) appear to be the best alternative, considering that the genetic correlations with its regressors and dry matter intake (DMI) are near zero. Residual CH4 is positively connected with residual feed intake (RFI), showing that animals producing lower CH_4_ also process feed more efficiently [163]. Another study used genome-wide association studies (GWAS) to examine the association of single nucleotide polymorphisms (SNPs) and genomic areas with eight CH_4_ emission variables in Danish Holstein cattle [163]. The traits studied were methane concentration (MeC; ppm), methane production (MeP; g/d), two definitions of residual methane (RMetc and RMetp: MeC and MeP regressed on metabolic body weight and energy-corrected milk, respectively), two definitions of methane intensity (MeI; MeIc = MeC/ECM and MeIp = MeP/ECM); two definitions of methane yield per kilogram of dry matter intake (MeY; MeYc = MeC/dry matter intake and MeYp = MeP/dry matter intake). There were significant relationships with three traits on chromosome 13 (MeC, MeP, and MeYc) and five traits on chromosome 26 (MeC, MeP, MeIp, MeYp, and MeYc). On chromosome 1, several intriguing connection signals were discovered for MeIc, MeIp, RMetc, MeYc, and MeYp. Based on their findings from GWAS and genetic correlations, scientists find that methane concentration is (genetically) more closely related to methane production than any of the other methane variables investigated [163]. Manzanilla et al. also showed that comparison to MeP, which just slows the pace of rising, including RMet in the breeding goal, would result in a true reduction in CH_4_ [163]. Other studies also talk about residual methane emission traits. The most prominent combination traits are ratio traits such as methane intensity (MeI; CH_4_ per kilogram of milk, milk yield, or ECM) and methane yield (MeY; CH_4_ per kg of DMI), as well as residual methane emission traits, which are estimated using multiple linear regression on various combinations of MBH, ECM, and DMI [164,165,166].

## 5. Conclusions

It is frequently stated that reducing CH_4_ emissions is a positive situation for the environment and livestock industry. Most studies on ruminant CH_4_ reductions related to food management are short-term and only look at changes in enteric emissions. All techniques to lowering enteric CH_4_ emissions should address the economic consequences on farm profitability and the linkages between enteric CH_4_ and other GHG, as many of the ones presented below are only partial approaches to reducing emissions. Although plant extracts work well in reducing methane emissions, sustainability must also be kept in mind, especially when it comes to sourcing and growing plants. The ability of plant components to reduce enteric CH_4_ emissions from ruminants depends on a number of factors, such as the amount of bioactive compound in the plant, which in turn depends on its availability and sustainability, as well as the methods used to harvest, transport, store, and process plants to make it into a feed ingredient. To ensure animal welfare and health, investigations on methane emission should be undertaken on a large number of animals over a long period, as well as the association of rumination duration that best represents the physiological state of ruminal fermentation at optimal levels. Mitigation methods are rarely used in vast grazing systems; however, nutritional management or the use of growth promoters can minimize methane output. It is possible that new natural chemicals that lower rumen methane emissions will be discovered in the future. There are more unsolved challenges. The safety of feeding algae (containing bromoform) to livestock must also be researched more closely, as also genetic selection according to promising traits. The incorporation of additives in ruminant diets must be made economical through improved producer prices for animal products and/or greater productivity resulting from optimizing animal nutrition for the methanogenesis inhibition intervention.

## 6. Future Directions

It is necessary to conduct additional research to fully understand the effects of methanogenesis inhibition on rumen fermentation and post-absorptive metabolism. The aim is to develop nutritional strategies that optimize the circulation of assimilated nutrients changed by the methanogenesis inhibition intervention to meet animal requirements and potentially improve animal productivity and efficiency.

## Figures and Tables

**Table 1:** Methods for measuring CH_4_ emissions.

Method	Short Elucidation
Respiration chambers (RC)	There are two types of RC: closed-circuit and open-circuit [25]. While closed-circuit systems are practically never used nowadays, open-circuit chambers are the most often exploited type, with varying degrees of complexity [25,47,48]. Individual animals are typically kept in chambers between 2 to 7 days, and CH_4_ emissions are estimated based on gas flow and changes in gas concentrations between the air coming in and out [10,25,48,49,50]. The chamber approach is expensive in terms of both investment and labor, and it has been accused of affecting feeding behavior. In trials employing transparent chambers, however, no impacts on dry matter intake (DMI) were observed [49]. However, only one cow may be tested at a time, and each test requires several hours in the respiration chamber, limiting research efficiency [10,18,25]. Almost in all studies, a single gas analyzer was used to measure in and out methane concentrations, often for two or more chambers [10,18,42]. When Garnsworthy et al. compared several different methods of GHG measuring, it was found that respiration chambers were the most accurate method. None of the correlations with other methods exceeded 0.90 [10].
Sulfur hexafluoride (SF_6_) tracer technique	The method is invasive—the cow must consume a bolus carrying the tracer, and the SF_6_ tracer is an exceptionally potent greenhouse gas [18,42]. The underlying premise is that the rate of SF_6_ gas release from the rumen is calculated in order to calculate the CH_4_ emission measurement [25,51]. The approach is suited for enclosed and free-roaming animals, and it involves inserting a permeation tube with a known SF_6_ gas release rate into the animal’s reticulorumen [49,52]. A tube hooked to a halter and connected to an evacuated canister worn around the animal’s neck or back is the basic premise behind this technique. Near the animal’s nostrils, the air is taken for testing purposes. It takes roughly 24 h for the canister to fill up between 50% and 70% because of an airflow restriction provided by a capillary tube. Methane emission rates are calculated by multiplying the predetermined SF_6_ release rate by the canister methane to SF_6_ concentration ratio [10,25]. The technique is more beneficial when evaluating CH_4_ emissions from individual animals. Wearing the device and daily handling to change canisters may affect animal behavior and feed intake. The sulfur hexafluoride (SF_6_) tracer technique is far less intrusive than breathing chambers because cows remain in the herd [42].
Spot sampling technique/ Gas-flux quantification system	The cornerstone of spot sampling approaches is the collection of acceptable short-term breath data for emission measurements. The techniques employ spot measurements of exhaled CH_4_ during milking or feeding. These procedures are typically automated, noninvasive, and non-intrusive, allowing for a high rate of animal throughput [45,49]. Breath sampling is taken during milking and feeding. The feed bin could be at a concentrate feeding station or an autonomous milking station [18,52,53]. These methods are referred to as “sniffer methods” since they use devices originally developed to detect harmful gas leaks. Air is sampled near the animal’s nostrils using a tube attached to a feed bin and immediately connected to a gas analyzer [10,18]. Methane concentrations measured during a sample visit of 3 to 10 min can be expressed as the overall mean or the mean of eructation peaks. Breath-sampling approaches provide substantial advantages over other methods for large-scale measurement of methane emissions by individual animals [20,25]. Some patented methods, such as the GreenFeed system, work in the same method as sniffer methods [25]. The GF method is based on the idea that a single animal’s daily average CH_4_ emission can be estimated by combining multiple short-term Methane emission measurements obtained throughout the day [25]. Breath-sampling procedures are noninvasive because animals are unaware of the apparatus and are in their natural environment after it is deployed. Animals continue their normal schedule, which includes milking and feeding; therefore, no animal training, handling, or dietary changes are required. Although more sophisticated gas analyzers are available, the equipment is quite inexpensive, and the operating costs are insignificant [20,45].
Carbon dioxide as a tracer to estimate daily methane emission	The methane and carbon dioxide ratio technique predicts CH_4_ output by certain species by forecasting carbon emissions and sensing methane and carbon dioxide concentrations [18,53]. This method demands knowledge about the ration’s consumption, energy content, and heat increase [54]. Using the CO_2_ technique does not consider the difference in CH_4_ emissions between efficient and inefficient cows; according to Huhtanen et al., Researchers found a strong correlation between the efficiency of low and high-efficiency cows. The technique overestimated the amount of CH_4_ produced by productive cows while underestimating the amount produced by ineffective cows [55]. Because it is so easy to apply to many animals, the standard error of means can be reduced [25].
Infrared ray spectroscopy, laser technique	Lasers have long been applied for gas detection in environmental monitoring, air quality monitoring, security, and health care [25]. Hand-held gas detectors for remote measurements of column density for methane-containing gases. It is based on infrared (IR) absorption spectroscopy. It uses a collimated semiconductor laser as an excitation source and wavelength modulation spectroscopy’s second harmonic detection to establish a methane concentration measurement [25,56]. Methane concentration measurements are performed manually using a portable instrument around 1–3 m away from the animal. The data acquisition sequence comprises small spans of 2–4 min. The resulting data is a series of peaks representing the animal’s breathing cycle [56,57]. The laser methane detector (LMD) can be used in the animal’s natural environment; however, a constraint is required during the measuring process to ensure accuracy. Results can be affected by factors such as the distance from the animal, the angle of pointing, the animal’s motion and moving direction, the airflow movement, and temperature in the barn since the LMD measures methane in the plume issuing from the animal’s nostrils [58].
Face mask (FM) method	The method for spot samplings of respiratory exchange and CH_4_ emissions is based on animals trained to remain in sternal recumbency for 30 min measurement periods taken every 2–3 h, with up to 7 measures per day [25]. In terms of assessing gas exchange and changes in the exhaled CH_4_ concentration, the basis of this method is identical to that of RC. It consists of a mass flow controller, gas sampling unit, and CH_4_ emission analyzer attached to each face mask. Gas readings are corrected for humidity, lag time, drift, and CH_4_ emission (mL/min) changes for each period [25,59]. The FM approach is less expensive and simpler than SF_6_ or RC. Its mobility allows it to measure multiple areas in order to collect CH_4_ emissions [25,60].

**Table 3:** Some of the methane-reducing supplements, their impact on animal health and sustainability.

Component	Methane Reducing Effect	Influence on Animal Health	Sustainability
Algae, bromoform	↓ 45–99% [23,37,98]	Bromoform can be excreted in urine and milk [99]. Weight increase was observed [23,100].	Seaweed raises water pH, hence mitigating ocean acidification (suitable habitat). Emits trace that degrades the ozone layer, dampens wave energy during storms, protects the coast, offers human consumption with biofuels, fertilizer, medicine, and food, animal food supplements [37,101,102,103].
Tannins	↓ 13–30% [104]	Increase total bacteria and fungi, decrease protozoa, and decrease methanogens [104,105,106,107]. Some decrease fungi but increase methanogens [108]. It can increase weight and production [109].	They are abundant in many plant species and may be extracted using simple procedures [109,110].
Saponins	↓ 7–23% [111]	Reduced populations of ruminal ciliate protozoa may accelerate microbial protein flow from the rumen, increasing feed utilization efficiency and decreasing methanogenesis [112]. Decrease protozoa, decrease methanogens [113].	They are eco-friendly due to their natural nature, biodegradable, and non-toxic, which is critical from an environmental and health standpoint. Saponins obtained from plants can be a sustainable alternative to synthetic surfactants [114,115].
Essential oils	↓ 8–22% [116]	Alternative feed booster, preserving mineral digestion [117]. Increasing MY, DMI, can improve milk fat and protein composition and decrease somatic cell count [116,118]. Improve the efficiency of microbial production [119].	When highly concentrated essential oils are used correctly, they may be both ecologically and economically sustainable.

## Data Availability

Not applicable.
